# A Retrospective Dosimetric Analysis of the New ESTRO-ACROP Target Volume Delineation Guidelines for Postmastectomy Volumetric Modulated Arc Therapy After Implant-Based Immediate Breast Reconstruction

**DOI:** 10.3389/fonc.2020.578921

**Published:** 2020-10-20

**Authors:** Kyung Hwan Chang, Jee Suk Chang, Kwangwoo Park, Seung Yeun Chung, Se Young Kim, Ryeong Hwang Park, Min Cheol Han, Jihun Kim, Hojin Kim, Ho Lee, Dong Wook Kim, Yong Bae Kim, Jin Sung Kim, Chae-Seon Hong

**Affiliations:** ^1^Department of Radiation Oncology, Yonsei Cancer Center, Yonsei University College of Medicine, Seoul, South Korea; ^2^Department of Radiation Oncology, Ajou University School of Medicine, Suwon, South Korea; ^3^Department of Radiation Oncology, Gangnam Severance Hospital, Yonsei University College of Medicine, Seoul, South Korea; ^4^Department of Radiation Oncology, Yongin Severance Hospital, Yonsei University College of Medicine, Yongin, South Korea

**Keywords:** breast cancer, ESTRO-ACROP guideline, immediate breast reconstruction, postmastectomy radiotherapy (PMRT), VMAT

## Abstract

**Purpose:** The European Society of Radiation & Oncology and Advisory Committee on Radiation Oncology Practice (ESTRO-ACROP) presented new guidelines for clinical target volume (CTV) delineation in post-mastectomy radiation therapy (PMRT) after implant-based immediate breast reconstruction (IBR-i). This study evaluated the dosimetric characteristics, dosimetric accuracy, and delivery accuracy of these guidelines in volumetric modulated arc therapy (VMAT).

**Methods and Materials:** This retrospective study included 15 patients with left breast cancer who underwent mastectomy with tissue expander placement followed by PMRT. An experienced radiation oncologist delineated the CTV twice on the same image datasets based on the ESTRO-ACROP (EA-TVD) and conventional target volume delineation (C-TVD) guidelines. All VMAT plans, which used a double partial arc, were generated using six MV photons. Clinically relevant dose-volume parameters for organs at risk were compared. Dosimetric accuracy of the treatment plans and delivery accuracy were assessed.

**Results:** Target volume of EA-TVD was significantly smaller than that of C-TVD. Although no statistically significant difference was noted in the target coverage between the two VMAT plans, EA-TVD VMAT significantly reduced the mean heart dose (3.99 ± 1.02 vs. 5.84 ± 1.78 Gy, *p* = 0.000), the maximum left anterior descending coronary artery (LAD) dose (9.43 ± 3.04 vs. 13.97 ± 6.04 Gy, *p* = 0.026), and the mean LAD dose (4.52 ± 1.31 vs. 6.35 ± 2.79 Gy, *p* = 0.028) compared with C-TVD VMAT. No significant difference was observed with respect to the total monitor units, plan complexity, and delivery quality assurance.

**Conclusions:** This is the first study to show significant dose reduction for the normal heart and LAD tissue offered by the EA-TVD, while maintaining dosimetric and delivery accuracy, in PMRT after IBR-i in VMAT for left-sided breast cancer patients.

## Introduction

In the United States and Europe, breast cancer is the one of the most common cancers, accounts for 30% of all new cancer diagnoses in women, and is the leading cause of cancer-related mortality in women worldwide (1–3).

Over the past two decades, the use of breast reconstruction has increased steadily (4, 5). In 2016, an estimated 109,250 women underwent breast reconstruction in the US, which was 39% more than the number in 2,000 (6). Breast reconstruction provides essential psychosocial, cosmetic, and quality of life benefits to patients with breast cancer who have undergone mastectomy (5, 7). A meta-analysis of patient data from 22 randomized trials conducted by the Early Breast Cancer Trialists' Collaborative Group showed that postmastectomy radiation therapy (PMRT) in patients with axillary dissection reduced breast cancer recurrence and mortality (8). Further, Miyashita et al. reported the decrease in locoregional recurrence of breast cancer due to PMRT in patients with 1–3 positive axillary lymph nodes (9).

Breast reconstruction methods in women receiving PMRT could be divided into three broad categories: based on the timing of reconstruction, immediate vs. delayed; type of reconstruction, implants vs. autologous; and timing of expander-to-implant exchange, before or after radiation therapy (RT) and the optimal time to perform exchange or delayed reconstruction following PMRT (10). The reconstruction is categorized as immediate, delayed, or delayed-immediate (10). There are two categories of the timing of expander-to-implant exchange, namely, single-stage and two-stage reconstruction. Single-stage reconstruction refers to the placement of a permanent prosthesis implant after mastectomy. Two-stage reconstruction refers to the placement of a tissue expander underneath the skin at the time of mastectomy; ~1 month after completion of chemotherapy, the tissue expander is exchanged for a permanent prosthesis (7, 11).

Although there has been an increase in the number of patients receiving PMRT, the use of PMRT following immediate breast reconstruction (IBR) is challenging. Current PMRT combined with IBR is often used as a field-based rather than a volume-based treatment for treatment field definition (12). This approach may detrimentally impact the target coverage and doses to the organs at risk (OAR) and is associated with an increased risk of complications (13). The use of modern volume-based PMRT, such as intensity-modulated RT (IMRT) or volumetric-modulated arc therapy (VMAT), can reduce treatment-related complications; however, consensus-based guidelines for target volumes in the setting of IBR have been insufficient.

Recently, the European Society of Radiation & Oncology and Advisory Committee on Radiation Oncology Practice (ESTRO-ACROP) introduced new guidelines for CTV delineation in the setting of PMRT after implant-based immediate breast reconstruction (IBR-i) by global multidisciplinary group experts (breast surgeon, plastic surgeon, radiation oncologists, and clinical oncologists) for breast cancer (13). The committee recommended target volume delineation (TVD) for the chest wall after pre-pectoral and retro-pectoral implantation (13). Although ESTRO-ACROP provided detailed guidelines on target volume definitions for PMRT in the setting of breast reconstruction, the guidelines do not provide sufficient details on the dosimetric analysis of RT planning. Several researchers have reported dosimetric results of PMRT using three-dimensional conformal RT (3DCRT), IMRT, and VMAT technique for breast cancer patients with expander reconstruction (14–17). However, to the best of our knowledge, there is no published report yet on the comparative dosimetric analysis of PMRT using VMAT plans based on the new ESTRO-ACROP guidelines for left-sided breast cancer patients.

This study aimed to evaluate and compare the dosimetric improvement, plan complexity, and delivery accuracy of ESTRO-ACROP guidelines-based (EA-TVD) and conventional guidelines-based (C-TVD) target volume delineation in PMRT using hypofractionated VMAT after IBR-i.

## Methods and Materials

### Patients Characteristics

Fifteen consecutive patients with left breast cancer who underwent mastectomy with tissue expander placement followed by PMRT with an intent to replace the expander to IBR on a later date were retrospectively included in this study. All patients were operated on and treated with immediate two-stage prosthetic reconstruction (all with retro-pectoral implants) between January 2017 and June 2019 (11). Two-stage breast reconstruction was performed in our institution as detailed in our previous study (11). In the first stage, the tissue expander was placed underneath the pectoralis muscle at the time of mastectomy. An expander was then filled incrementally with saline for 2 weeks after the first operation and partially deflated before PMRT. Approximately 3 months after the completion of RT, the tissue expander was exchanged with a permanent implant (11). The average target volume of EA-TVD and C-TVD were 440.7 ± 108.3 and 775.6 ± 153.8 cm^3^, respectively (Table 1).

**Table 1:** Comparison of target coverage for VMAT plans between the EA-TVD and C-TVD guidelines.

**Parameters**	**EA-TVD**	**C-TVD**	***p*-value**
CTV volume (cm^3^)	440.7 ± 108.3	775.6 ± 153.8	0.000*
V_95_ (%)	94.3 ± 3.9	95.7 ± 1.6	0.121
V_105_ (%)	4.5 ± 7.9	6.9 ± 10.1	0.505
D_5_ (Gy)	41.8 ± 1.8	42.0 ± 0.6	0.668
D_95_ (Gy)	37.5 ± 1.6	38.2 ± 0.6	0.071
D_mean_ (Gy)	39.9 ± 1.0	40.4 ± 0.4	0.074
nCI	0.7 ± 0.8	0.8 ± 0.6	0.714
HI	1.1 ± 0.1	1.1 ± 0.0	0.273

ESTRO-ACROP, European Society of Radiation & Oncology Advisory Committee on Radiation Oncology Practice; EA-TVD, ESTRO-ACROP target volume delineation; C-TVD, Conventional target volume delineation; CTV, clinical target volume; V_D_, percentage volume receiving D% of the prescribed dose; D_V_, the dose covering V% of the target; D_mean_, mean dose; nCI, new conformity index; HI, homogeneity index. ^*^Indicates a statistically significant difference.

### Image Acquisition and Volume Definition

All patients underwent computed tomography (CT) scanning (Siemens Healthineers, Germany) in a supine position with 3 mm slice thickness in free breathing. The acquired CT images were transferred to MIM (Version 6.5.6; MIM Software Inc., Cleveland, OH, United States), which was used to contour CTV and OAR. A single expert radiation oncologist contoured the CTV twice to compare the dosimetric characteristics between the two TVD guidelines.

**Figure 1:**
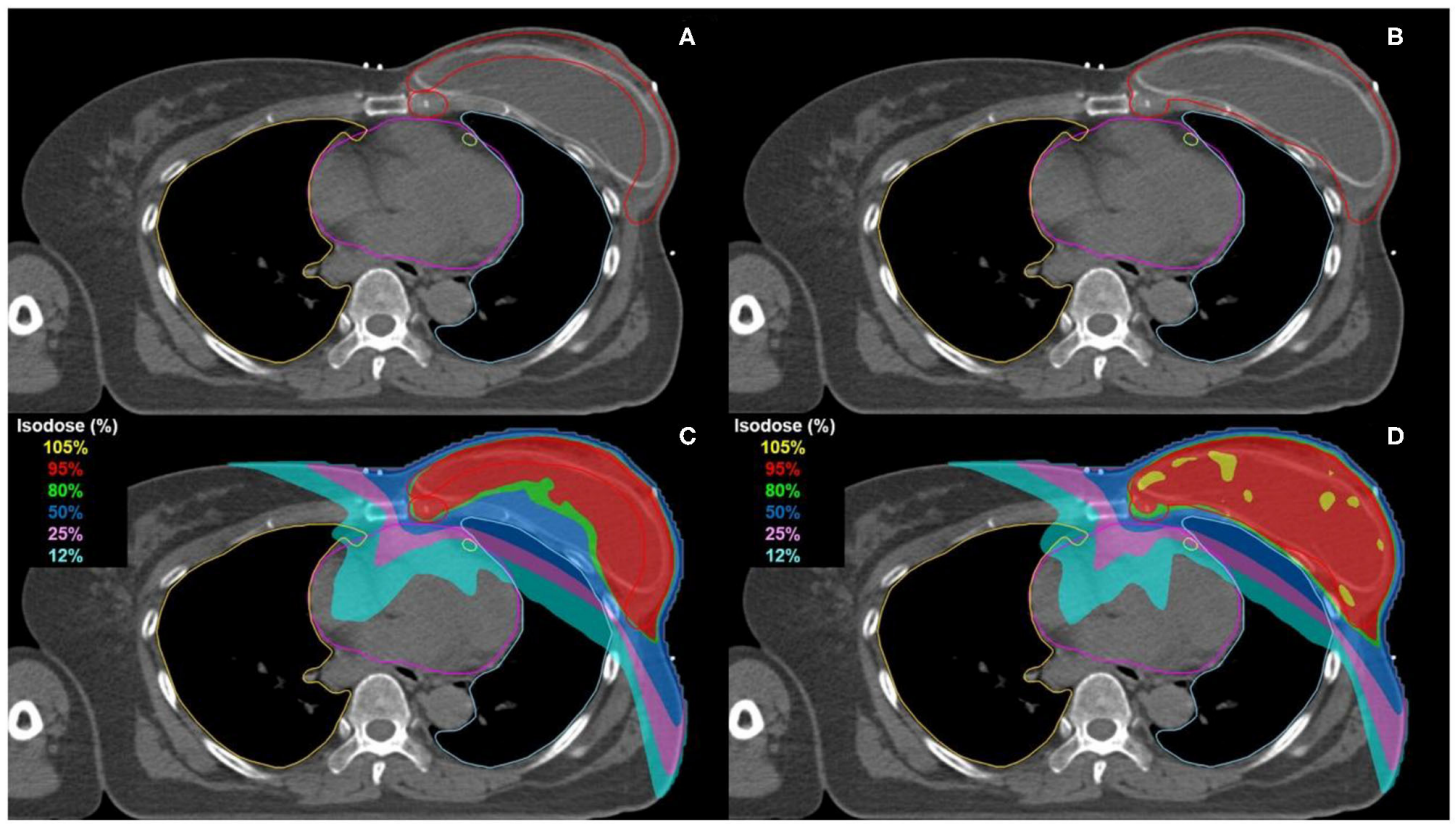
Comparison of CTV delineation and dose distribution on a transversal slice for representative patient. Delineation of CTV was performed according to the EASRTO-ACROP consensus guideline **(A)** and the conventional guideline **(B)**. The CTV, heart, LAD, ipsilateral lung, and contralateral lung are delimited in red, pink, light green, light blue, and orange, respectively. The 105, 95, 80, 50, 25, and 12% isodose of the prescribed dose (40.05 Gy) are represented in yellow, red, green, blue, pink, and cyan, respectively **(C,D)**.

The CTV was delineated including the ipsilateral chest wall and regional lymph nodes in all patients. It was defined as “EA-TVD” for the ESTRO-ACROP guidelines-based and as “C-TVD” for the conventional guidelines-based target volume delineation. The delineation of regional lymph nodes, except the chest wall, was consistent between the two delineation guidelines. For EA-TVD, the CTV of chest wall was contoured according to the ESTRO-ACROP TVD guidelines in case of the retro-pectoral implants. The dorsal part of the chest wall CTV included the major pectoral muscles or ribs and the intercostal muscles. While the ventral part of the chest wall is always part of the CTV, the dorsal part is only included depending on the anatomical and tumor-related risk factors. If the tumor was localized in areas within the breast close to the dorsal fascia not covered with the major pectoral muscle, the ESTRO-ACROP guideline recommended the delineation of the tissue between the chest wall and the implant caudal from the pre-surgical position of the major pectoral muscle that can be performed as a separate dorsal CTV. This detailed contouring guideline has been described in previous studies (13). For the C-TVD, the CTV of the chest wall was contoured with reference to the ESTRO target volume guidelines (18). The CTV delineation based on ESTRO-ACROP (Figure 1A) and conventional (Figure 1B) guidelines on a transversal slice of a representative patient is illustrated in Figure 1.

The heart, left anterior descending coronary artery (LAD), ipsilateral and contralateral lung, and contralateral breast were contoured according to the RTOG recommendations (19). All contours were then imported into the RayStation treatment planning system (TPS, v5.0.2.35, RaySearch Laboratories, Stockholm, Sweden) for VMAT planning.

### Treatment Planning

For each patient, a double coplanar arc VMAT plan was generated on the RayStation. A linear accelerator of 6 MV photon beam (Versa HD, Elekta, UK) was used in this study. Arc 1 usually starts at 295–305° and stops at 155–165° while Arc 2 is rotated in the reverse direction as that of Arc 1. The collimator angles are 0° for Arc 1 and 15° for Arc 2. For hypofractionated VMAT, the prescribed dose was 40.05 Gy in 15 fractions of 2.67 Gy to the CTV. All plans were normalized so that at least 95% of the chest wall CTV received 95% of the prescribed dose, and the planning criteria for planning target volume (PTV) coverage and dose to normal organ were based on the institution's practice guidelines (11). The maximum dose to any point was limited to 105%, particularly in CTV, to minimize the occurrence of a hotspot. Normal tissue constraints were as follows: maximum and mean dose of the contralateral breast were <5 and 2 Gy, respectively; heart mean dose <5 Gy; maximum and mean dose of LAD <12 and 5 Gy, respectively; ipsilateral lung volume receiving 5 Gy (V_5Gy_), 10 Gy (V_10Gy_), and 20 Gy (V_20Gy_) were limited to 50, 35, and 20%, respectively; mean dose of contralateral lung <2–3 Gy. Moreover, if possible, the dose to normal tissues (especially heart and LAD) was kept as low as possible while maintaining the PTV dose. For all VMAT plans, dose calculation was performed using a collapsed cone algorithm with a dose grid size of 2 mm in the RayStation. All the treatment plans for the two groups were performed with two dosimetrists, and each dosimetrist was blinded to any information about the treatment plan results according to the patients.

### Dosimetric Comparison

All dose-volume histograms were extracted and evaluated for all the targets and OARs in the study. With respect to the CTV, the volume receiving 95 and 105% of the prescribed dose (V_95_ and V_105_), average dose delivered to the CTV (D_mean_), and dose covering 95% (D_95_) and the most exposed 5% (D_5_) of the target were compared. To evaluate target coverage, the homogeneity index (HI) and new conformity index (nCI) were calculated for CTV in all plans. The HI and nCI were calculated as follows:

$$\begin{array}{l} \text{HI}={\text{D}}_{5\text{\%}}/{\text{D}}_{95\text{\%}},\text{nCI}=\left({\text{PTV}}_{\text{PIV}}^{2}\right)/\left(\text{PTV }\times \text{ PIV}\right), \end{array}$$

where PTV_PIV_ is the PTV encompassed within the prescription isodose volume (PIV), which is the volume covered by the prescription isodose surface (20, 21). An HI value of 1 is the ideal value that indicates uniform dose distribution within the target, and a CI value of 1 is indicative of perfect conformation. V_5Gy_, V_10Gy_, V_20Gy_, V_30Gy_, V_40Gy_, mean dose (D_mean_), and maximum dose (D_max_) were calculated for the heart, where V_DGy_ represents the percentage volume of structures receiving at least D Gy of radiation dose. Further, D_max_ and D_mean_ were calculated for the LAD; V_5Gy_, V_10Gy_, V_20Gy_, V_40Gy_, and D_mean_ were calculated for the ipsilateral lung; V_5Gy_, V_10Gy_, V_20Gy_, and D_max_ were calculated for the contralateral lung; and D_mean_ was calculated for the contralateral breast.

### Plan Complexity and Delivery Quality Assurance (DQA)

Plan complexity was evaluated to analyze the dosimetric accuracy by quantifying the degree of modulation according to the change in the shape of the CTV (inverted *U*-shaped vs. hemisphere-shaped) when adopting EA-TVD instead of C-TVD in the hypofractionated VMAT. For all plans in both groups, plan complexity was analyzed using the modulation index (MI), which was calculated using the algorithm developed previously (22). An increase in the MI value indicates that beam modulation is complexed. In addition, the total monitor units (MUs) for all plans in both groups were analyzed.

DQA was performed using the MapCHECK (Model 1082, Sun Nuclear, Melbourne, FL, United States) detector on each plan to evaluate the dose calculation and delivery accuracy. We calculated the dose difference (DD) and gamma passing rate (GPR) between planned and measured doses in two delineation guidelines using the SNC Patient software (Version 6.4.1., Sun Nuclear, Melbourne, FL, United States). The point dose for the DD was measured in the absolute dose mode at the isocenter position, and the DD and distance-to-agreement acceptance criteria for the global gamma analysis were 3% and 3 mm, respectively.

### Statistical Analysis

A paired two-tailed *t-*test was performed to calculate the *p*-value (SPSS, version 25, Chicago, IL, United States). In this study, *p* ≤ 0.05 were considered statistically significant.

## Results

### Dosimetric Evaluation

Table 1 summarizes the quantitative dosimetric analysis for the target volume. The mean target volume of EA-TVD (440.7 ± 108.3 cm^3^) was significantly smaller than that of C-TVD (775.6 ± 153.8 cm^3^; *p* < 0.001). There were no significant differences in V_95_, V_105_, D_5_, D_95_, D_mean_, nCI, and HI, in terms of the various dosimetric parameters associated with target coverage of CTV between the two TVD guidelines.

**Figure 2:**
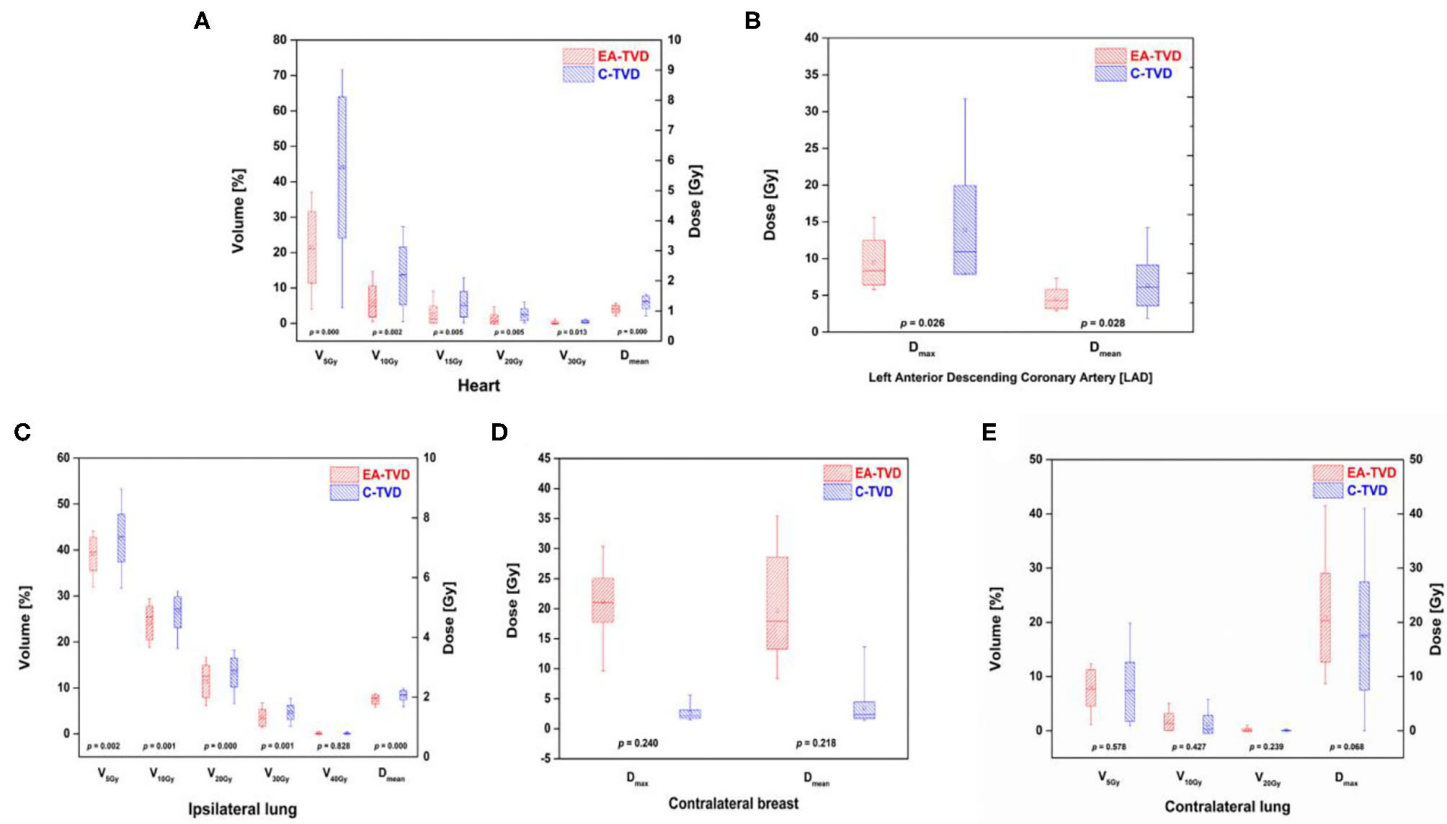
Box plots of all OARs dose volume indices for the ESTRO-ACROP target volume delineation (EA-TVD) and conventional target volume delineation (C-TVD). The box plots of the EA-TVD and C-TVD plan for each OAR are shown in red and blue, respectively. **(A)** heart, **(B)** left anterior descending coronary artery (LAD), **(C)** ipsilateral lung, **(D)** contralateral breast, and **(E)** contralateral lung.

Table 2 presents the dosimetric comparisons of OAR between EA-TVD and C-TVD, whereas the dose distribution for a representative patient is illustrated in Figures 1C,D. The V_5Gy_, V_10Gy_, V_20Gy_, V_30Gy_, and D_mean_ values of the heart in the EA-TVD plan were significantly lower than those in the C-TVD plan (Figure 2A). The EA-TVD plan led to clearly lower doses in D_max_ and D_mean_ of the LAD than the C-TVD plan (Figure 2B). A significantly better sparing of the V_5Gy_ to V_30Gy_, mean heart dose (EA-TVD 4.0 ± 1.0 vs. C-TVD 5.8 ± 1.8 Gy, *p* < 0.000), maximum LAD dose (EA-TVD 9.4 ± 3.0 vs. C-TVD 14.0 ± 6.0 Gy, *p* = 0.026), and mean LAD dose (EA-TVD 4.5 ± 1.3 vs. C-TVD 6.4 ± 2.8 Gy, *p* = 0.028) with a more than 40% reduction in average dose was achieved with EA-TVD. Furthermore, with the EA-TVD, a statistically significant reduction in V_5Gy_, V_10Gy_, V_20Gy_, V_30Gy_, and D_mean_ of the ipsilateral lung was noted than in the C-TVD (*p* ≤ 0.002; Figure 2C). No statistically significant differences were observed in the contralateral lung and breast, between the two TVD guidelines (*p* > 0.05; Table 2, Figures 2D,E).

**Table 2:** Comparison of the organs at risk for VMAT plans between the EA-TVD and C-TVD guidelines.

**OARs**	**Index**	**EA-TVD**	**C-TVD**	***p*-value**
Heart	V_5Gy_ (%)	21.5 ± 10.3	42.7 ± 20.0	0.000*
	V_10Gy_ (%)	6.2 ± 4.4	12.8 ± 8.2	0.002*
	V_15Gy_ (%)	2.4 ± 2.4	5.2 ± 3.6	0.005*
	V_20Gy_ (%)	1.1 ± 1.3	2.3 ± 1.7	0.005*
	V_30Gy_ (%)	0.2 ± 0.4	0.4 ± 0.4	0.013*
	V_40Gy_ (%)	0.0 ± 0.0	0.0 ± 0.0	0.789
	D_mean_ (Gy)	4.0 ± 1.0	5.8 ± 1.8	0.000*
LAD	D_max_ (Gy)	9.4 ± 3.0	14.0 ± 6.0	0.026*
	D_mean_ (Gy)	4.5 ± 1.3	6.4 ± 2.8	0.028*
Ipsilateral lung	V_5Gy_ (%)	39.1 ± 3.6	42.7 ± 5.2	0.002*
	V_10Gy_ (%)	24.1 ± 3.7	26.7 ± 3.4	0.001*
	V_20Gy_ (%)	11.4 ± 3.5	13.7 ± 3.2	0.000*
	V_30Gy_ (%)	3.5 ± 1.8	4.9 ± 1.5	0.001*
	V_40Gy_ (%)	0.1 ± 0.1	0.1 ± 0.1	0.828
	D_mean_ (Gy)	7.5 ± 1.0	8.5 ± 1.0	0.000*
Contralateral breast	D_max_ (Gy)	21.2 ± 5.7	19.8 ± 8.7	0.240
	D_mean_ (Gy)	2.6 ± 1.3	3.3 ± 3.1	0.218
Contralateral lung	V_5Gy_ (%)	7.9 ± 3.4	6.8 ± 5.4	0.578
	V_10Gy_ (%)	1.6 ± 1.6	1.1 ± 1.7	0.427
	V_20Gy_ (%)	0.1 ± 0.3	0.0 ± 0.1	0.239
	D_max_ (Gy)	20.9 ± 8.2	17.0 ± 10.0	0.068

ESTRO-ACROP, European Society of Radiation & Oncology Advisory Committee on Radiation Oncology Practice; EA-TVD, ESTRO-ACROP target volume delineation; C-TVD, Conventional target volume delineation; CTV, clinical target volume; V_DGy_, percentage volume of a given structure receiving a radiation dose of D Gy; D_mean_, mean dose; D_max_, maximum point dose. *Indicates a statistically significant difference.

### Plan Complexity and Delivery Accuracy

Table 3 summarizes the comparison of MI, DQA results, and total MUs between the two TVD guidelines. The average MI was 9.1 ± 1.1 and 9.4 ± 1.3 for EA-TVD and C-TVD, respectively, and no statistically significant differences were observed between the two guidelines (*p* = 0.451). In addition, the C-TVD plan had a lower mean total MU than the EA-TVD plan. However, this difference was not statistically significant (*p* = 0.213). The average DD values were 0.2 ± 2.5% for EA-TVD and −0.2 ± 1.5% for C-TVD (*p* = 0.602), while the average GPRs were 94.3 ± 2.0 and 95.2 ± 1.9% for EA-TVD and C-TVD, respectively (*p* = 0.267). Finally, no statistically significant differences were noted in the DQA results between the two guidelines.

**Table 3:** Comparison of the modulation index, DQA results, and total MUs for VMAT plans between the EA-TVD and C-TVD guidelines.

**Index**	**EA-TVD**	**C-TVD**	***p*-value**
Modulation Index	9.1 ± 1.1	9.4 ± 1.3	0.451
Dose difference [%]	0.2 ± 2.5	−0.2 ± 1.5	0.602
Gamma passing rate [%]	94.3 ± 2.1	95.2 ± 1.9	0.267
Total MUs	1106.1 ± 277.8	1020.4 ± 259.9	0.213

*ESTRO-ACROP, European Society of Radiation & Oncology Advisory Committee on Radiation Oncology Practice; EA-TVD, ESTRO-ACROP target volume delineation; C-TVD, Conventional target volume delineation; MU, monitor unit*.

## Discussions

Although there has been an increase in the implementation of PMRT after breast reconstruction, there is a lack of consensus guidelines on target volume definition and RT planning. ESTRO-ACROP has recently provided detailed guidelines on target volume definitions, although even these guidelines do not provide sufficient details on the dosimetric analysis of RT planning (13). In this study, we compared the dosimetric characteristics, treatment plan complexity, and delivery accuracy in VMAT treatment plans between the target delineation method used in our institution (EA-TVD) and the C-TVD including the whole breast.

As shown in Table 1, there was no significant difference in the target coverage between the two groups. Since the EA-TVD had an inverted *U*-shaped target, the homogeneity and conformity of the target were expected to be lower than that of the conventional contouring method. However, we confirmed that there was no statistically significant difference between the two groups.

The new EA-TVD provided a significantly lower dose to the OAR, especially to the heart and LAD for left breast cancer patients. LAD sparing may have an advantage in EA-TVD due to the inverted U-shaped target, as shown in Figure 1, allowing the target to be physically separated from the LAD.

Several studies have reported the association of cardiac toxicity with radiation for breast cancer (23–27). Darby et al. conducted a study on 2,168 patients who received breast radiotherapy and showed that the rates of major coronary events increased linearly with the mean dose to the heart by almost 7.4% per Gy (24). Similarly, Van de Bogaard et al. studied the relationship between acute coronary events and mean heart dose in 910 breast cancer patients and confirmed that the incidence of acute coronary event increased by ~16.5% per Gy of mean heart dose. In this study, researchers noted that it is important to reduce the dose delivered to the heart to avoid the risk of coronary events after RT in breast cancer patients (25). Skytta et al. evaluated the effect of left-sided breast cancer RT on serum high-sensitivity troponin T (hscTnT) levels and its association with cardiac doses (26). The authors reported that an increase in radiation doses in patients with left-sided breast cancer could lead to subclinical myocardial damage; thus, it is necessary to make efforts to maintain the radiation dose of the heart as low as possible (26). In the present study, the mean heart dose in the EA-TVD was about 2 Gy less than that in the conventional contouring method (3.99 vs. 5.84 Gy, *p* < 0.000). This suggests that VMAT using the EA-TVD guidelines can potentially reduce the incidence of coronary events after PMRT after IBR-i by approximately 15–33% as opposed to the conventional contouring method, which includes the entire chest wall with the implant.

The deep inspiration breath holding (DIBH) and VMAT are reportedly useful strategies for minimizing cardiac toxicity associated with left-side breast cancer radiotherapy. Several researchers have reported that DIBH with VMAT significantly decreased the mean heart dose and LAD dose in contrast to free breathing (FB) with VMAT (17, 27–29). Kuo et al. evaluated the effect of DIBH and VMAT for locally advanced breast cancer patients with expander or implant reconstruction receiving comprehensive PMRT. The comparative study found that the mean heart dose was 7.5 ± 1.1 Gy and 6.6 ± 0.8 Gy for FB and DIBH, respectively, and the maximum dose to the LAD was 33.8 ± 11.7 Gy and 31.4 ± 7.3 Gy for FB and DIBH, respectively (17). Further, Sakka et al. showed that there was a significant decrease in the mean heart dose (5.3 to 4.03 Gy) and the mean LAD dose (8.7 to 7.3 Gy) between DIBH with VMAT and FB with VMAT (27). Corradinie et al. reported that the mean heart dose was 2.4 ± 0.7 Gy and 2.9 ± 1.4 Gy for VMAT with DIBH and FB, respectively, when four different RT techniques were evaluated in 10 left-sided early stage breast cancer cases (28). In yet another study, Dumane et al. showed that the mean heart dose and maximum dose to the LAD were reduced by 2.9 Gy (8.2 to 5.3 Gy) and 9.9 Gy (40.7 to 30.8 Gy), respectively, for DIBH with VMAT in left-sided breast cancer patients with implant reconstruction receiving regional nodal irradiation (29). In this study, the use of EA-TVD reduced the mean heart dose, mean LAD dose, and maximum LAD dose by 1.9, 1.8, and 4.5 Gy, respectively (Table 2). Based on these results, we confirmed that VMAT planning using the EA-TVD guidelines can achieve cardiac and LAD dose reduction similar to that achieved on combining VMAT and DIBH. Therefore, we are planning to investigate the dose reduction effect on the heart and LAD for VMAT plans involving a combination of DIBH and EA-TVD guidelines.

We analyzed the plan complexity, DQA results, and total MU to evaluate the dosimetric accuracy, delivery accuracy, and efficiency of the treatment plan as the target shape changed to a more complex shape. However, as shown in Table 3, when compared with C-TVD, the delivery accuracy and efficiency of the treatment plan using the EA-TVD did not deteriorate, and there was no statistically significant difference in the homogeneity of the target.

The application of hypofractionated regimens has recently increased, and they have a reportedly local control equivalent to that of the conventional fraction regimen. Chang et al. suggested that some hypofractionated regimens have the potential to reduce complications in the setting of breast reconstruction (11). In the present study, VMAT plans were generated using a hypofractionated regimen, and our results show that this hypofractionated VMAT technique along with the EA-TVD guidelines has dosimetric benefits while maintaining delivery accuracy and efficiency. However, further studies are needed to investigate the clinical effects of heart and LAD dose reduction in these hypofractionated methods.

This study has several limitations inherent to retrospective studies. The EA-TVD guidelines recommend the exclusion of the tissue between the chest wall and the implant beneath the presurgical position of the pectoralis major in high-risk cases such as locally advanced breast cancer and residual disease after preoperative chemotherapy (30). First, this study was only focused on the evaluation of dosimetric characteristics, delivery accuracy, and efficiency for EA-TVD guidelines. Therefore, we could not evaluate the effect of the EA-TVD guidelines on the majority of clinically detected chest wall recurrences that occur in the skin and subcutaneous tissues (30, 31). Second, the dosimetric benefits presented in this study are results specific to the VMAT. 3DCRT and IMRT, which are most commonly used in clinical practice, were not included in this study. PMRT combined with IBR should generally include treatment of the internal mammary nodes (IMNs) that drain the breast (32, 33). In this case, although IMN irradiation may show a survival benefit, conventional radiotherapy is difficult to perform without compromising normal tissue toxicity (i.e., the heart, lung, and contralateral breast) or target coverage (13, 33). Therefore, more advanced planning techniques are indispensable, and VMAT is an effective way to reduce the dose to the heart and the ipsilateral lung and improve treatment outcomes compared to the conventional technique (34–36). Meanwhile, these advantages come with the cost of low-dose spread to the contralateral tissues, i.e., the contralateral breast and the lung, raising concerns about a potential increase of secondary cancer risk. Hence, many radiation oncologists are still reluctant to employ IMRT (or VMAT) for PMRT patients; however, several studies have reported that the risk of secondary cancer in IMRT is similar to that of 3DCRT (36–38). Consequently, the choice of optimal planning technique for PMRT should be based on the consideration of the balance between all relevant risks, e.g., normal tissue toxicity and radiation-induced secondary cancer risk. In this study, VMAT was applied to patients with left-sided breast cancer undergoing regional nodal irradiation who were unable to meet the dose constraints in the heart and lungs with 3DCRT (11, 39). In addition, dose constraints for the contralateral breast were restricted to remain as low as possible, to minimize (D_mean_ <3 Gy) the risk of contralateral breast cancer due to the low-dose spread (40). Third, the robustness of the VMAT plans with respect to setup and respiratory motion uncertainties was not considered in this study. The use of a precise treatment technique, such as VMAT, creates dosimetric changes even with small uncertainties.

## Conclusions

The ESTRO-ACROP consensus guidelines describe delineation of target volume in PMRT after IBR-i. This is the first study that confirms the dosimetric characteristics, dosimetric accuracy, and delivery accuracy of these guidelines in VMAT and demonstrates their dosimetric benefits, especially at lower doses to both the heart and the LAD, when compared with C-TVD. No statistically significant differences were observed in dosimetric and delivery accuracy between the two TVD guidelines. Our results provide evidence of the dosimetric advantages of EA-TVD, which will help radiation oncologists determine the clinical application of the EA-TVD guidelines.

## Data Availability Statement

The data analyzed in this study is subject to the following licenses/restrictions: The datasets analyzed during the current study are available from the corresponding authors on reasonable request. Requests to access these datasets should be directed to Chae-Seon Hong, cs.hong@yuhs.ac.

## Ethics Statement

The studies involving human participants were reviewed and approved by Yonsei University College of Medicine, Yongin Severance Hospital, Institutional Review Board (9-2020-0069). Written informed consent for participation was not required for this study in accordance with the national legislation and the institutional requirements.

## Author Contributions

KC, C-SH, JC, and JSK drafted the manuscript and worked on the conception, design, and interpretation of data. SC, SK, and RP performed the radiation therapy plan. KP, MH, JK, HK, HL, DK, and YK reviewed the data analysis. All authors have approved the final manuscript.

## Conflict of Interest

The authors declare that the research was conducted in the absence of any commercial or financial relationships that could be construed as a potential conflict of interest.
